# Birth outcomes and survival by sex among newborns and children under 2 in the Birhan Cohort: a prospective cohort study in the Amhara Region of Ethiopia

**DOI:** 10.1136/bmjgh-2024-015475

**Published:** 2024-08-13

**Authors:** Emily Thompson, Getachew Mullu Kassa, Robera Olana Fite, Clara Pons-Duran, Frederick G B Goddard, Alemayehu Worku, Sebastien Haneuse, Bezawit Mesfin Hunegnaw, Delayehu Bekele, Kassahun Alemu, Lisanu Taddesse, Grace J Chan

**Affiliations:** 1 Department of Biostatistics, Harvard University T H Chan School of Public Health, Boston, Massachusetts, USA; 2 Health System and Reproductive Health Research Directorate, EPHI, Addis Ababa, Ethiopia; 3 HaSET Maternal and Child Health Research Program, Addis Ababa, Ethiopia; 4 Department of Epidemiology, Harvard University T H Chan School of Public Health, Boston, Massachusetts, USA; 5 Department of Pediatrics and Child Health, Saint Paul's Hospital Millennium Medical College, Addis Ababa, Ethiopia; 6 Department of Obstetrics and Gynecology, Saint Paul's Hospital Millennium Medical College, Addis Ababa, Ethiopia; 7 Department of Pediatrics, Boston Children's Hospital, Harvard Medical School, Boston, Massachusetts, USA

**Keywords:** Cohort study, Global Health, Child health

## Abstract

**Introduction:**

Despite the progress in reducing child mortality, the rate remains high, particularly in sub-Saharan African countries. Limited data exist on child survival and other birth outcomes by sex. This study compared survival rates and birth outcomes by sex among neonates and children under 2 in Ethiopia.

**Methods:**

Women who gave birth after 28 weeks of gestation and their newborns were included in the analysis. Survival probabilities were estimated for males and females in the neonatal period as well as the 2-year period following birth using Kaplan-Meier curves. HRs and 95% CIs were compared between males and females under 2. Descriptive statistics and χ^2^ tests were used to determine the sex-disaggregated variation in the birth outcomes of preterm birth, low birth weight (LBW), stillbirth, small for gestational age (SGA) and large for gestational age (LGA).

**Results:**

The study included a total of 3904 women and child pairs. The neonatal mortality rate for males (3.4%, 95% CI 2.6% to 4.2%) was higher compared with females (1.7%, 95% CI 1.1% to 2.3%). The hazard of death during the first 28 days of life was approximately two times higher for males compared with females (HR 1.99, 95% CI 1.30 to 3.06) but was not significantly different after this period. While there was a non-significant difference between males and females in the proportion of preterm, LBW and LGA births, we found a significantly higher proportion of stillbirth (2.7% vs 1.3%, p=0.003) and SGA (20.5% vs 15.6%, p<0.001) for males compared with females.

**Conclusions:**

This study identified a significant sex difference in mortality and birth outcomes. We recommend focusing future research on the mechanisms of these sex differences in order to better design intervention programmes to reduce disparities and improve outcomes for neonates.

**WHAT IS ALREADY KNOWN ON THIS TOPIC**

- In low-income and middle-income countries, the burden of infant morbidity and mortality is especially high. Fetal sex impacts health during and after pregnancy, but studies comparing birth outcomes by sex are limited and oftentimes contradictory.

**WHAT THIS STUDY ADDS**

- In the Birhan Cohort, we found a higher rate of neonatal mortality for male compared with female babies, but this disparity subsides after the neonatal period. Male babies also had higher proportions of stillbirths and small for gestational age births than female babies.

**HOW THIS STUDY MIGHT AFFECT RESEARCH, PRACTICE OR POLICY**

- This study contributes to the literature on sex-based differences in infant outcomes and suggests, particularly in Ethiopia and similar settings, that targeted interventions that take these sex-based differences into account may be effective.

## Background

 Globally, much progress has been made to reduce child mortality; in 2020, 1 in 27 children died before the age of 5, compared with 1 in 11 children in 1990.^1^ While global health initiatives have prevented millions of deaths, under-5 mortality remains a significant problem, with 13 800 under-5 deaths occurring every day in 2020.^2^ The first 28 days of life (the neonatal period) are a particularly vulnerable period in child development, especially in low-income and middle-income countries (LMICs); more than half (53.1%) of under-5 deaths in LMICs occur during the neonatal period while 98% of all neonatal mortality occurs in LMICs.^3 4^

One factor that can impact mortality and morbidity is fetal sex, which has an effect on maternal and child health during and after pregnancy.^5^ Although not clearly understood, the sex differences in child health outcomes could be influenced by biological variation at the cellular and molecular level among male and female fetuses.^6^ Such variations are affected by maternal characteristics including maternal glucocorticoids, health status during pregnancy and maternal prepregnancy overweight or obesity status.^6^ Some studies have also shown that the average crown-rump length and biparietal diameter, which are indicators of fetal development and are associated with morbidity, were higher in male fetuses than in female fetuses at the measurement during the first trimester.^7^,^9^ Furthermore, the difference in placental function could influence fetal growth and sex-specific differences in child health outcomes.^6^

Limited studies have shown significant differences in the distribution of neonatal outcomes between male and female neonates.^5^ For example, the odds of fetal heart rate patterns and low Apgar score at 5 min were significantly higher among male than female neonates.^10^ Although some findings are inconsistent, there appear to be sex differences in adverse birth outcomes like small for gestational age (SGA), large for gestational age (LGA), preterm birth, low birth weight (LBW) and stillbirth.^11^,^19^ In addition, previous studies have suggested that male babies in Ethiopia and other LMICs experience higher mortality rates than females due in part to a higher risk for males of morbidities such as prematurity, respiratory infections and intrauterine growth restriction; however, these studies have not investigated the timing of this survival disparity.^20 21^

We conducted this study to estimate the relationship between sex and mortality among children less than 2 years of age in the Amhara region of Ethiopia. We further investigated the sex differences in birth outcomes including preterm birth, LBW, stillbirth, SGA, LGA and aetiologies of mortality. Data were collected from the Birhan Cohort, which includes longitudinal follow-up during the first 2 years of life, so under-2 mortality was the focus of this study. The findings of the study will guide the development of programmes and interventions for the prevention of neonatal morbidity and mortality in Ethiopia. Furthermore, sex-disaggregated data provide evidence for programme managers and policy-makers on sex-disaggregated neonatal morbidity and mortality.

## Methods

### Study setting and design

This prospective cohort study was conducted in the Birhan field site in Ethiopia. The Birhan field site was established in 2018 and includes a health and demographic surveillance system (HDSS), the Birhan HDSS, a platform for community-based and facility-based research with a focus on maternal and child health.^22^ It is located in the North Shewa Zone, Amhara Regional State, Ethiopia with over 82.6% of the population living in rural areas. The zone is located 130 km north of Addis Ababa, the capital city of Ethiopia. The Birhan field site includes 16 villages from Angolela Tera and Kewet districts and the Shewa Robit city administration.^22^ There were 18 933 households and 77 766 individuals enrolled in the Birhan HDSS as of mid-2019.

The Birhan field site includes a nested open pregnancy and birth cohort, the Birhan Cohort, to assess exposure variables and their association with maternal and child health outcomes, enrolling approximately 2000 pregnant women and their newborns per year with rigorous longitudinal follow-up over the first 2 years of life and household data linked with health facility information.^23^ In this study, we used data from pregnant women enrolled in the Birhan Cohort from December 2018 to August 2023 and their children.

### Data collection procedure

Women were enrolled in the Birhan Cohort at different time points and could be enrolled during the pregnancy, labour, delivery or postpartum period. Women living in the Birhan catchment area were screened for pregnancy every 3 months and could be identified for enrollment at the community level. Women could also be identified and enrolled in the study when at a facility for antenatal care (ANC) visit, delivery or postnatal care visit. Postpartum women were also followed immediately after birth, at days 6, 28 and 42. For the child, data were collected at birth, at each postnatal visit with the mother, and 6, 12 and 24 months after birth. Data were collected using an electronic data collection system and were cleaned and audited for quality assurance before analysis.^23^ To determine probable causes of neonatal mortality, we used data collected by verbal autopsy (VA).^24 25^

### Eligibility criteria

Women enrolled in the Birhan Cohort who delivered between December 2018 and August 2023 and their newborn babies were included in the study because stillbirths in Ethiopia are defined as occurring at or after 28 weeks and a gestational age of 46 weeks or greater is considered implausible, only deliveries between 28 weeks and 45 weeks of gestation were included.^26^ Women who were enrolled in the Birhan Cohort but had a miscarriage, abortion or delivery before 28 weeks of gestation were excluded from the study.^26^

### Study measures and variable definitions

Data used for primary study measures were collected during scheduled home visits and facility visits for ANC or delivery. Home visits were conducted every 3 months until 32 weeks of gestation, every 2 weeks from 32 weeks to 36 weeks of gestation and every week from 36 weeks of gestation through delivery. Gestational age was estimated following a hierarchy based on availability and reliability of data (ultrasound measurement, last menstrual period and/or fundal height).^26^ Birth weight was measured using Seca 354 digital scales.^26^

The primary study measures were mortality, stillbirth, SGA, LGA, preterm birth and LBW. We defined SGA as a live birth weight of less than the 10th percentile for gestational ages between 28 and 43 weeks and LGA as a live birth weight above the 90th percentile, based on Intergrowth-21 standards.^26^,^28^ Preterm birth was defined as a live birth occurring at less than 37 completed weeks of gestation.^29^,^31^ LBW was defined as a live birth with birth weight less than 2500 g, excluding birth weights above the 99th percentile.^26 29 32 33^ Stillbirth was defined as a birth with no signs of life like heartbeat or non-zero Apgar score occurring at or after 28 weeks of gestational age.^26 34^ Finally, we defined neonatal mortality as the death of a neonate during the first 28 completed days of life and under-2 mortality as the death of an infant during the first 2 years of life.^35^

To assess sociodemographic characteristics of the cohort, we examined maternal age at conception, marital status, employment status, education level, district of residence and wealth quintile (using an index created based on living conditions and assets). To assess obstetric characteristics, we examined gravidity (total number of previous pregnancies), parity (total number of previous pregnancies resulting in a live or stillbirth), previous miscarriage or stillbirth, previous twin birth, mode of delivery and delivery type, and ANC visit attendance. Baseline obstetric variables were collected during enrolment in the Birhan Cohort and self-reported demographic variables were collected and updated during house-to-house surveillance every 3 months. ANC attendance was initially determined through self-report at enrolment. Following enrolment, data on ANC visits were prospectively gathered from facility charts.^36^

### Data processing and analysis

Maternal and child characteristics were presented using descriptive statistics including frequencies and summary statistics (median, IQR and percentage).

Mortality rates were estimated for males and females over the first 2 years of life using Kaplan-Meier estimation. We then fit a Cox regression model with the male-female HR varying over time, specifically within the following windows: 0–28 days, 29–60 days and 61–730 days. These time intervals were chosen based on the hypothesis that survival differences would be most prominent in the first few months of life. From these, time interval-specific HR estimates and 95% CIs were estimated.^37^

Note that we expect the independent censoring assumption to hold and for Kaplan-Meier estimates to be unbiased (ie, we expect the mortality rate of those who were censored to be the same as those who completed follow-up). Data collection through the HDSS includes house-to-house rounds that update cases of mortality that were previously missed. Additionally, outmigration is a common cause of loss to follow-up that is expected to affect infants equally.

Descriptive analyses were used to determine the sex-disaggregated variation in the causes of neonatal mortality. Sex differences in the non-mortality birth outcomes defined above were quantified using Pearson’s χ^2^ tests for differences in proportions at a Bonferroni-corrected significance level of 0.01 for five hypothesis tests.

Data cleaning and analysis were conducted using STATA software (V.17) and R-software (V.4.2.2).

### Patient and public involvement

The Birhan field site involves a community advisory board, which meets regularly to contribute to the activities conducted in the Birhan Cohort. The field site engages with the public through the community advisory board.

## Results

A total of 3904 woman and child pairs were included in this study, 3825 (98.0%) of them live births. Births with a gestational age of less than 28 weeks (42 woman-child pairs, 1.0%) and greater than 45 weeks (22 woman-child pairs, 0.6%) were excluded, giving the final sample of 3904. Of the included births, 1897 newborns were female (48.6%) and 2007 were male (51.4%). Multiple pregnancies in the same individual were treated as separate births; these accounted for 112 (2.9%) of births.

Of the 3904 woman and child pairs in the study, 366 (9.4%) out-migrated from the field site sometime during the 2 years following delivery. Out of 2116 births that reached the age of two by the end of August 2023 (the cut-off to be included in the study) and were not known to have out-migrated or died, 974 (46.0%) did not complete their 24-month visit. See online supplemental table 1 for the count of study participants by year.

### Sociodemographic characteristics

Maternal sociodemographic characteristics were similar for male and female babies (table 1). The median maternal age at conception was 27.0 (IQR: 22.6–31.1) years. The majority of babies were born to mothers who were married or living with a partner, and about 55% of mothers had a primary or higher level of education. Just over half of mothers resided in the Kewet/Shewa Robit district. About 40% of babies were born to women in the two lowest quintiles of wealth, while almost 35% of babies were born to women in the two highest quintiles.

**Table 1:** Distribution of sociodemographic characteristics of respondents by neonatal sex

	All births (N=3904)	Female (N=1897)	Male (N=2007)
Mother age at conception			
Median (IQR)	27.0 (22.6–31.1)	27.0 (22.9–31.2)	26.7 (22.2–31.0)
Missing	14 (0.4%)	6 (0.3%)	8 (0.4%)
Marital status			
Divorced or widowed	66 (1.7%)	28 (1.5%)	38 (1.9%)
Married or living with a partner	3489 (89.4%)	1705 (89.9%)	1784 (88.9%)
Unmarried	339 (8.7%)	159 (8.4%)	180 (9.0%)
Missing	10 (0.3%)	5 (0.3%)	5 (0.2%)
Occupation			
Employed	3701 (94.8%)	1793 (94.5%)	1908 (95.1%)
Unemployed	175 (4.5%)	88 (4.6%)	87 (4.3%)
Missing	28 (0.7%)	16 (0.8%)	12 (0.6%)
Education level			
None	1152 (29.5%)	547 (28.8%)	605 (30.1%)
Primary	1750 (44.8%)	841 (44.3%)	909 (45.3%)
Secondary and above	421 (10.8%)	206 (10.9%)	215 (10.7%)
Missing	581 (14.9%)	303 (16.0%)	278 (13.9%)
District			
Angolela Tera	1765 (45.2%)	860 (45.3%)	905 (45.1%)
Kewet/Shewa Robit	2133 (54.6%)	1035 (54.6%)	1098 (54.7%)
Missing	6 (0.2%)	2 (0.1%)	4 (0.2%)
Wealth quintile			
Poorest	817 (20.9%)	386 (20.3%)	431 (21.5%)
Poorer	843 (21.6%)	414 (21.8%)	429 (21.4%)
Medium	740 (19.0%)	382 (20.1%)	358 (17.8%)
Richer	677 (17.3%)	325 (17.1%)	352 (17.5%)
Richest	650 (16.6%)	300 (15.8%)	350 (17.4%)
Missing	177 (4.5%)	90 (4.7%)	87 (4.3%)

### Obstetric characteristics

Maternal obstetric characteristics were also similar for male and female babies (table 2). About half of babies were born to multigravida and multipara women. The majority of women did not have a previous miscarriage or stillbirth and did not have a previous twin birth. The most common mode of delivery was vaginal delivery for about 70% of births, and 80% had cephalic vertex presentation. Around 80% of mothers of both male and female babies had one or more ANC visits, although only about 20% had complete ANC visits of four or more.

**Table 2:** Distribution of obstetric characteristics of respondents by sex of the newborn

	All births (N=3904)	Female (N=1897)	Male (N=2007)
Gravidity			
Multigravida	2064 (52.9%)	1018 (53.7%)	1046 (52.1%)
Primigravida	785 (20.1%)	394 (20.8%)	391 (19.5%)
Missing	1055 (27.0%)	485 (25.6%)	570 (28.4%)
Parity			
Multipara	1864 (47.7%)	929 (49.0%)	935 (46.6%)
Primipara	835 (21.4%)	412 (21.7%)	423 (21.1%)
Nullipara	1181 (30.3%)	544 (28.7%)	637 (31.7%)
Missing	24 (0.6%)	12 (0.6%)	12 (0.6%)
Previous miscarriage or stillbirth			
None	2256 (57.8%)	1126 (59.4%)	1130 (56.3%)
One or more	575 (14.7%)	276 (14.5%)	299 (14.9%)
Missing	1073 (27.5%)	495 (26.1%)	578 (28.8%)
Previous twin birth			
No	1744 (44.7%)	860 (45.3%)	884 (44.0%)
Yes	101 (2.6%)	53 (2.8%)	48 (2.4%)
Missing	2059 (52.7%)	984 (51.9%)	1075 (53.6%)
Mode of delivery			
C-Section	196 (5.0%)	89 (4.7%)	107 (5.3%)
Vaginal delivery	2844 (72.8%)	1371 (72.3%)	1473 (73.4%)
Missing	864 (22.1%)	437 (23.0%)	427 (21.3%)
Vaginal delivery type			
Breech	52 (1.3%)	22 (1.2%)	30 (1.5%)
Cephalic vertex	3097 (79.3%)	1501 (79.1%)	1596 (79.5%)
Face and other	755 (19.3%)	374 (19.7%)	381 (19.0%)
Any ANC visit			
None	724 (18.5%)	346 (18.2%)	378 (18.8%)
One or more ANC visit	3180 (81.5%)	1551 (81.8%)	1629 (81.2%)
ANC visit completeness			
Complete ANC (4 or more visits)	738 (18.9%)	371 (19.6%)	367 (18.3%)
Incomplete ANC (<4 visits)	3166 (81.1%)	1526 (80.4%)	1640 (81.7%)

ANC, antenatal care.

### Survival analysis

**Figure 1:**
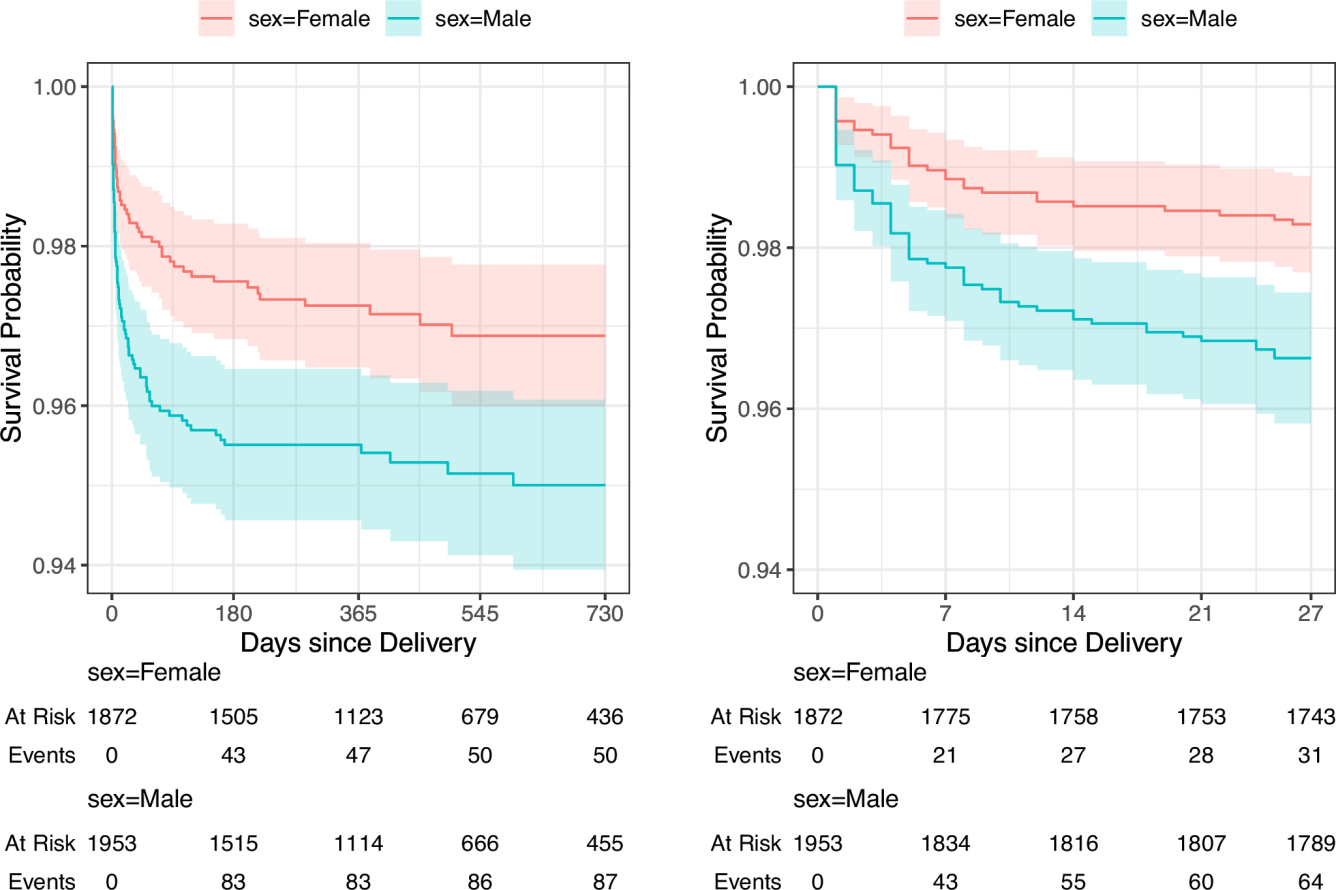
Kaplan-Meier survival curve of under-2 mortality (left) and neonatal mortality (right) by sex. Shaded regions represent 95% CIs.

Among 3825 live births, there were 95 neonatal deaths and 137 deaths by age 2, with 64 (67.4%) of the neonatal deaths and 87 (63.5%) of the deaths by age 2 being among males. The Kaplan-Meier estimates of mortality in the first 28 days were 1.7% (95% CI 1.1% to 2.3%) for females and 3.4% (95% CI 2.6% to 4.2%) for males; estimates of mortality in the first 2 years were 3.1% (95% CI 2.2% to 4%) for females and 5.0% (95% CI 3.9% to 6.1%) for males. The survival curve of neonatal mortality shows that females had a higher survival probability than males immediately following birth while the survival curve of deaths by age 2 shows that differences in survival between males and females occurred during the neonatal phase and survival probabilities remained relatively consistent between males and females from 28 days to 2 years (figure 1). We observed consistent patterns of mortality for births in both the pre-COVID-19 and post-COVID-19 time periods, but more follow-up time would be needed to fully power separate analyses.

During the neonatal period, males had twice the hazard of death occurring in the day 0 to day 28 period that females did (HR 1.99, 95% CI 1.30 to 3.06) while the HRs between males and females in later time periods were not significantly different (table 3).

**Table 3:** HR of death occurring (male compared with female) by time period after delivery

	HR	95% CI
Time period (days since delivery)		
0–28 days	1.99	1.30 to 3.06
29–60 days	2.71	0.86 to 8.50
61 days to 2 years	0.80	0.38 to 1.71

CI, confidence interval; HR, hazard ratio.

Note that, because it is not clear what factors are simultaneously associated with infant sex and survival, we did not adjust for confounders in this analysis.

### Causes of neonatal death

The three most common primary causes of neonatal mortality were birth asphyxia, neonatal sepsis and prematurity. 54% of male neonatal deaths were caused by prematurity compared with 26.1% of female neonatal deaths. A larger proportion of female neonatal deaths were caused by sepsis (30.4%) compared with males (7.0%) while males and females experienced birth asphyxia in similar proportions (about 40%) (online supplemental table 2).

### Other adverse neonatal outcomes

There were 576 preterm births (14.8%) and 79 stillbirths (2.0%) among 3904 births. A similar proportion of male and female births were preterm, but the proportion of stillbirths among female babies was about half the proportion among male babies (1.3% of females vs 2.7% of males). Male and female babies were LBW and LGA in similar proportions (about 7%), but fewer females than males were SGA (15.6% of females vs 20.5% of males) (table 4).

**Table 4:** Sex differences in non-mortality neonatal outcomes

	All births (N=3904)	Female (N=1897)	Male (N=2007)	P value
Preterm birth				0.957
No	3249 (83.2%)	1589 (83.8%)	1660 (82.7 %)	
Yes	576 (14.8%)	283 (14.9%)	293 (14.6%)	
Not applicable (stillbirth)	79 (2.0%)	25 (1.3%)	54 (2.7%)	
Stillbirth				0.00338*
No	3825 (98.0%)	1872 (98.7%)	1953 (97.3%)	
Yes	79 (2.0%)	25 (1.3%)	54 (2.7%)	
LBW				0.705
No	2875 (73.6%)	1383 (72.9%)	1492 (74.3%)	
Yes	285 (7.3%)	141 (7.4%)	144 (7.2%)	
Missing	744 (19.1%)	373 (19.7%)	371 (18.5%)	
SGA				<0.001*
No	2298 (58.9%)	1158 (61.0%)	1140 (56.8%)	
Yes	708 (18.1%)	296 (15.6%)	412 (20.5%)	
Missing	898 (23.0%)	443 (23.4%)	455 (22.7%)	
LGA				0.613
No	2730 (69.9%)	1316 (69.4%)	1414 (70.5%)	
Yes	276 (7.1%)	138 (7.3%)	138 (6.9%)	
Missing	898 (23.0%)	443 (23.4%)	455 (22.7%)	

* Significant differences between the proportion of males and females with the outcome at a significance level of 0.01.

LBW, low birth weight; LGA, large for gestational age; SGA, small for gestational age.

Birth weight was missing for 665 live births (17.4%), which prevented classification of LBW, SGA and LGA; missingness was approximately equally prevalent for both males (317 births, 8.1%) and females (348 births, 8.9%).

## Discussion

In this study, we estimated the effect of sex on neonatal and under-2 mortality and other birth outcomes. We found that neonatal mortality was significantly higher in males than females while differences between male and female survival after the neonatal period diminished. We also identified a non-significant difference in the proportion of LBW, preterm and LGA births. However, there was a significantly higher proportion of SGA and stillbirths among males than female newborns. The leading cause of neonatal mortality among male newborns was prematurity and among females was birth asphyxia.

Our finding that neonatal survival is higher for females than males is consistent with other studies of Ethiopia and other LMICs.^20 21^ Possible explanations for this include the higher risks of intrauterine growth restriction, prematurity, respiratory distress syndrome and birth asphyxia among male compared with female infants.^21 38^

While we found a non-significant difference in the proportion of preterm birth among male and female newborns, several studies suggest a higher risk of preterm birth for male newborns, but findings are mixed.^11^,^16^ Similarly, we found a non-significant difference in the proportion of LBW among male and female newborn babies while previous reports have indicated a higher incidence of LBW among female than male babies.^39 40^ We also found a significant difference in the proportion of stillbirth among male (2.7%) and female neonates (1.3%); this is consistent with some previous research,^17 18^ but other studies have suggested that stillbirth is higher among females compared with males.^19^ Similarly to our results, previous studies have shown a higher risk of SGA for males than females.^17^ While neonatal sex appears to play a role in birth outcomes, it is clear that this is a complex issue influenced by a variety of factors. Despite this complexity, it is important to identify sex-specific disparities in birth outcomes and mortality rates in order to design targeted interventions aimed at reducing these adverse effects, especially in resource-limited settings.

Some of the interventions recommended by the WHO to improve child health and reduce mortality include strengthening care provided at birth or during the first week of life, improving the quality of maternal and neonatal healthcare services before and after delivery, and expanding care provided to newborns with diseases.^41^ We found that complete ANC visit coverage (defined as four or more visits) was lacking in our cohort, with only around 20% of study participants having complete coverage. This is an important finding to address in future work as ANC coverage is associated with better birth outcomes and lower risk of neonatal mortality.^42^,^44^ These interventions, along with targeted sex-specific programmes to reduce the disparities identified in this paper, could greatly reduce mortality and morbidity among children and infants in Ethiopia.

While many of our findings in this paper are consistent with prior work, there are some limitations of the study. For instance, we estimated gestational age using a hierarchy of best available data including ultrasound gestational age measurements to minimise the risk of incorrect estimation, but maternal recall of the last menstrual period can have low accuracy due to recall bias. There was also a high percentage of missing (17%) birth weight measurements in our data. In addition, 46% of babies that reached the age of two during the study did not complete their 24-month follow-up visit, which could be explained in part by COVID-19 pauses in data collection and regional conflicts during this time period. These issues also led to a pause in enrolment of pregnant women between April 2020 and January 2022. While we expect the missingness not to significantly bias the analysis presented here, as male and female births were missing birth weight in similar proportions and missing visits or enrolments due to data collection issues are not expected to be associated with either sex of the baby or birth outcomes, more complete data would improve the precision of estimates of outcomes and strengthen conclusions.

## Conclusions

This study revealed sex differences in the proportions of adverse newborn and neonatal outcomes, with significant differences observed in the proportion of stillbirth, mortality and SGA outcomes. These outcomes were more prevalent among male babies compared with female babies. Among males, prematurity was the primary cause of death, while birth asphyxia was the leading cause among females. The survival curve of neonatal mortality further highlighted these sex-based differences, showing that female neonates had a higher survival probability than males, with their survival curves diverging immediately following birth; these survival differences diminished in the postneonatal period.

These findings underscore the need for future large-scale studies to further investigate the relationship between the sex of the baby and additional neonatal and maternal outcomes in Ethiopia and similar global settings. They also highlight the importance of considering sex-based variations when designing intervention programmes aimed at preventing stillbirth, neonatal mortality, early childhood mortality and SGA. Furthermore, to better understand the biological mechanisms underlying the sex-based variations in newborn and neonatal outcomes identified in this study, future studies are recommended. Such research could provide valuable insights that could help improve neonatal health outcomes and reduce mortality and morbidity rates.

## Supplementary material

10.1136/bmjgh-2024-015475 online supplemental file 1

10.1136/bmjgh-2024-015475 online supplemental file 2

## Data Availability

Data are available on reasonable request.
