# Supplementary material for: Birth outcomes and survival by sex among newborns and children under 2 in the Birhan Cohort: a prospective cohort study in the Amhara Region of Ethiopia
Source: BMJ Glob Health. 2024 Aug 13;9(8):e015475. doi: 10.1136/bmjgh-2024-015475 (PMC11331882; doi:10.1136/bmjgh-2024-015475)
Supplement: online supplemental file 1 [file bmjgh-9-8-s001.pdf]

Supplemental Table 1. Number of study participants by year (December 2018 through August 2023). Note that the COVID-19 pandemic and ongoing conflicts in Ethiopia led to a pause in the enrollment of pregnant women between April 2020 and January 2022.

**Year of delivery**	**Number of woman-child pairs**
2018	41
2019	1552
2020	925
2021	0
2022	947
2023	439

Supplemental Table 2. The sex-disaggregated difference in the primary cause of neonatal mortality using data from Verbal Autopsy (VA)

	**Overall (N=66)**	**Female (N=23)**	**Male (N=43)**
Top three primary causes of death			
Birth asphyxia	27 (40.9%)	10 (43.5%)	17 (39.5%)
Neonatal sepsis	10 (15.2%)	7 (30.4%)	3 (7.0%)
Prematurity	29 (43.9%)	6 (26.1%)	23 (53.5%)
