# Supplementary material for: Birth outcomes and survival by sex among newborns and children under 2 in the Birhan Cohort: a prospective cohort study in the Amhara Region of Ethiopia
Source: BMJ Glob Health. 2024 Aug 13;9(8):e015475. doi: 10.1136/bmjgh-2024-015475 (PMC11331882; doi:10.1136/bmjgh-2024-015475)
Supplement: online supplemental file 2 [file bmjgh-9-8-s002.docx]

# **Reflexivity Statement**

This study is part of the HaSET Maternal and Child Health Research Program, a partnership of Harvard T.H. Chan School of Public Health, Boston Children’s Hospital, St. Paul’s Hospital Millennium Medical College, and the Ethiopian Public Health Institute^[[1]](#footnote-1)^. The team includes epidemiologists, clinicians, public health professionals and policy makers based in Ethiopia and the United States. HaSET’s research is based in the Birhan field site, in North Shewa zone, Ethiopia.

1. **How does this study address local research and policy priorities?**

This study addresses research and policy priorities that are aligned with the mission of our research program, and that are also among the top priorities of the Ministry of Health in Ethiopia: to advance the health of mothers, newborns and children by generating quality evidence and translating results to programs and policies. The team is in close contact with the Ethiopian Ministry of Health and the regional health systems to tackle their priorities with the studies performed in the Birhan field site.

1. **How were local researchers involved in study design?**

The study design of the Health and Demographic Surveillance System (HDSS), and the pregnancy-child cohort was co-led by the researchers in Ethiopia and the United States (US). Dr Grace J. Chan (PI) and Dr Dilayehu Bekele (co-PI) led the design and publication of the study protocols^[[2]](#footnote-2),^^[[3]](#footnote-3)^. Dr Bezawit Mesfin Hunegnaw is the Research Manager, and she is responsible for the overall supervision of the data collection and data management teams; she also contributed to the study design of the HDSS and the cohort.

The present secondary analysis of the Birhan data was led by two researchers in partnership, Emily Thompson in the US and Getachew Mullu Kassa in Ethiopia, and supervised by Dr. Grace J. Chan, with input from local researchers. While five of the co-authors are affiliated with U.S. institutions, seven are local researchers in Ethiopia.

1. **How has funding been used to support the local research team?**

This project was funded by the Bill and Melinda Gates Foundation through different investments, one of them being a Fellowship to train early career professionals (postdoctoral and implementation fellows) in Ethiopia. The other investments fund the data collection, data management, and analysis of the data collected. Most of the resources are allocated to the local research team.

1. **How are research staff who conducted data collection acknowledged?**

DB and BMH, who led and supervised data collection, are authors of this manuscript. Data collectors and field supervisors are acknowledged as a group in the Acknowledgements section.

1. **Do all members of the research partnership have access to study data?**

All team members with research-related roles were included in the study protocol for ethical review approval, and then granted access to the study data. All authors of this paper had access to study data.

1. **How was data used to develop analytical skills within the partnership?**

The data collected as part of the Birhan HDSS and pregnancy-child cohort is used to develop analytical skills of different team members both in Ethiopia and the US. Early career professionals and research analysts are mentored by more senior researchers, and analyses are reviewed by a fellow team member to ensure quality and capacity building.

1. **How have research partners collaborated in interpreting study data?**

Study design, progress of analysis and results interpretation were discussed on a weekly basis in analysis meetings. All research partners were invited to participate in those meetings.

1. **How were research partners supported to develop writing skills?**

Co-lead authors (ET and GMK) who led the writing of the first draft of the manuscript received active support and feedback from all other research partners involved in the study.

1. **How will research products be shared to address local needs?**

The results of this study may contribute to resource allocation in resource-limited settings in general, and in the Birhan field site in particular. First, we plan to publish this paper as open access to get the results available for a broader audience for free. Then, we are also planning to share the study results with local stakeholders such as the Ministry of Health or the health authorities of the study area for them to use those to make policy decisions.

**10. How is the leadership, contribution and ownership of this work by LMIC researchers recognised within the authorship?**

GMK is a co-lead author of this work, and was responsible for the formulating the study question, the study design, and writing the manuscript.

DB is the co-PI of the research program. He contributed to obtaining funding for the study, led the data collection team, participated in data collection activities, critically discussed the study results, reviewed the manuscript, and approved the final version of the manuscript.

BMH coordinated the data collection and data management team, participated in data collection activities, critically discussed the study results, reviewed the manuscript, and approved the final version of the manuscript.

**11. How have early career researchers across the partnership been included within the authorship team?**

ET, CPD, BW and FGBG are early career researchers from both the US and Ethiopia. They were in charge of leading the study design and analysis (ET) and discussing results and reviewing the manuscript (CPD, BW, FGBG). They were supported and mentored by other authors of this manuscript such as the study PIs (GJC and DB), and the senior statistician (SH).

**12. How has gender balance been addressed within the authorship?**

Four authors, including one of the co-lead authors, are female (ET, CPD, BMH, GJC) and the remaining eight are male. The HaSET team has both female and male researchers leading different sub-studies and analyses.

**13. How has the project contributed to training of LMIC researchers?**

Overall, the Birhan HDSS and pregnancy cohort is a platform that trains early career researchers in Ethiopia through a funded Fellowship. This specific secondary analysis was co-led by GMK from Ethiopia and he contributed to the study design and manuscript development.

**14. How has the project contributed to improvements in local infrastructure?**

Part of the funding received for the HDSS and the pregnancy-child cohort was used to acquire materials and tools such as ultrasound machines, weighting scales are mid-upper arm circumference (MUAC) tapes to improve the follow-up of pregnant women and their children living in the study area.

**15. What safeguarding procedures were used to protect local study participants and researchers?**

To protect local study participants and researchers, all field related activities are led and implemented by local researchers. The HaSET team always makes decisions that have an impact on study participants and local team based on the advice, experience, and knowledge of the team members in Ethiopia. Cultural norms and local priorities are always taken into account before a decision is made.

1. <https://www.hsph.harvard.edu/haset/> [↑](#footnote-ref-1)
2. <https://bmjopen.bmj.com/content/11/9/e049692> [↑](#footnote-ref-2)
3. <https://academic.oup.com/ije/article/51/2/e39/6424216> [↑](#footnote-ref-3)
